# Relationship Between Population Density, Availability of Gynecological Services, and Cervical Cancer Incidence and Mortality Across Administrative Units in Serbia and Bosnia and Herzegovina During 2016–2020

**DOI:** 10.3390/medicina60121987

**Published:** 2024-12-02

**Authors:** Vladimir Vuković, Mirjana Štrbac, Mioljub Ristić, Siniša Skočibušić, Šeila Cilović-Lagarija, Jela Aćimović, Slađana Šiljak, Snežana Živković Perišić, Nataša Nikolić, Stefan Ljubičić, Tatjana Pustahija, Smiljana Rajčević, Aleksandra Patić, Tihomir Dugandžija, Aljoša Mandić, Vladimir Petrović

**Affiliations:** 1 Institute of Public Health of Vojvodina, 21000 Novi Sad, Serbia; mirjana.strbac@izjzv.org.rs (M.Š.); mioljub.ristic@mf.uns.ac.rs (M.R.); natasa.nikolic@mf.uns.ac.rs (N.N.); stefan.ljubicic@izjzv.org.rs (S.L.); tatjana.pustahija@mf.uns.ac.rs (T.P.); smiljana.rajcevic@mf.uns.ac.rs (S.R.); aleksandra.patic@mf.uns.ac.rs (A.P.); vladimir.petrovic@izjzv.org.rs (V.P.); 2 Faculty of Medicine, University of Novi Sad, 21000 Novi Sad, Serbia; tihomir.dugandzija@mf.uns.ac.rs (T.D.); aljosa.mandic@mf.uns.ac.rs (A.M.); 3 Institute for Public Health of the Federation of Bosnia and Herzegovina, 71000 Sarajevo, Bosnia and Herzegovina; s.skocibusic@zzjzfbih.ba (S.S.); s.cilovic@zzjzfbih.ba (Š.C.-L.); 4 Faculty of Medicine, University of Mostar, 88000 Mostar, Bosnia and Herzegovina; 5 Public Health Institute of the Republic of Srpska, 78000 Banja Luka, Bosnia and Herzegovina; jela.acimovic@gmail.com (J.A.); sladjana.siljak@phi.rs.ba (S.Š.); 6 Faculty of Medicine, University of Banja Luka, 78000 Banja Luka, Bosnia and Herzegovina; 7 Institute of Public Health of Serbia “Dr Milan Jovanović Batut”, 11000 Belgrade, Serbia; snezana_zivkovic@batut.org.rs; 8 Oncology Institute of Vojvodina, 21204 Sremska Kamenica, Serbia

**Keywords:** uterine cervical neoplasms, incidence, mortality, demography, health services, public health, Eastern Europe, Serbia, Bosnia and Herzegovina

## Abstract

*Background and Objectives*: Burden of cervical cancer in Central and Eastern Europe is higher than in other parts of Europe. We analyzed cervical cancer epidemiology in Serbia and Bosnia and Herzegovina (the Federation of Bosnia and Herzegovina and the Republic of Srpska) from January 2016 to December 2020, exploring the role of available sociodemographic factors and healthcare service parameters on incidence and mortality rates, using an ecological approach based on aggregated data. *Materials and Methods*: Incidence and mortality rates are standardized using the method of direct standardization with the World-ASR-W. Administrative units are grouped by tertiles of incidence and mortality to explore sociodemographic factors and healthcare parameters across these groups. *Results*: Average age-standardized incidence rates of cervical cancer per 100,000 females were 19.28 in Serbia, 12.48 in the Federation of Bosnia and Herzegovina, and 22.44 in the Republic of Srpska. Mortality rates per 100,000 females were 6.67, 5.22, and 4.56 in Serbia, the Federation of Bosnia and Herzegovina, and the Republic of Srpska, respectively. Several parameters of sociodemographics and health service usage differed significantly across units grouped by tertiles based on incidence level, i.e., female population ≥ 15 years old (*p* = 0.028), population density (*p* = 0.046), percent of gynecologists in the primary healthcare (*p* = 0.041), number of gynecologists per 10,000 females ≥ 15 years (*p* = 0.007), and the area-to-gynecologist ratio (*p* = 0.010). A moderate negative correlation was found between incidence and population density (rho = −0.465, *p* = 0.017), and a moderate positive correlation between incidence and area-to-gynecologist ratio (rho = 0.534, *p* = 0.005). *Conclusions*: Cervical cancer remains a leading cause of cancer among women in developing countries. Implementing tailored activities, such as educational programs, preventive services, and investments in healthcare infrastructure, particularly at the administrative units’ level, can help in reducing health disparities and improving health outcomes.

## 1. Introduction

According to recent estimates, there are more than 2.97 billion women of reproductive age worldwide who are at risk of developing cervical cancer [1]. Approximately 604,127 women are diagnosed with cervical cancer annually, and 341,831 died from this disease in 2020. Overall, cervical cancer ranks as the fourth most frequent cancer in women globally, based on the crude incidence rates [2]. The burden of cervical cancer is not evenly distributed; over 90% of cervical cancer deaths occur in low- and middle-income countries, with the highest incidence and mortality rates registered in sub-Saharan Africa, Central America, and South-East Asia [3]. In Europe, nearly 328 million women are at risk of developing cervical cancer, with an estimated 58,169 diagnosed and 25,989 dying from the disease each year. Cervical cancer ranks as the ninth leading cause of female cancer in Europe overall (according to crude incidence rates), but among women aged 15 to 44 years, it is the third most common cancer [4].

The burden of cervical cancer in Central and Eastern Europe is markedly higher than in other parts of Europe [5]. This elevated burden may be attributed to the effects of the post-socialist transition over the last three decades, which has impacted health-related issues and healthcare policies, including disease prevention [5,6]. Serbia is considered a high-risk region for cervical cancer—it is the fifth most common cancer among women and the second leading type of cancer for those aged 15 to 44 years [2]. The mortality rate is high, significantly exceeding the Southern European average as well as the global average [6,7]. In the neighboring Bosnia and Herzegovina, cervical cancer is ranked as the sixth most frequent in the country and the second most common cancer among women aged 15 to 44 years [8].

Persistent infection with the oncogenic strain of HPV is crucial for the development of precancerous lesions and cervical cancer. A recent study found that 43% of 10,062 women tested in Vojvodina (Serbia) over a 10-year period were HPV-positive, with the most common high-risk HPV genotypes being 16, 31, 52, 56, 39, and 51, including cases of multiple infections [9]. This highlights the urgent need for HPV vaccination, as highlighted by the WHO Cervical Cancer Elimination Strategy [10]. Countries with extensive HPV vaccination programs and high coverage of HPV vaccination have reported significant reductions in HPV infections, genital warts, and cervical precancerous lesions, with expected future decreases in cervical and other HPV-related cancers [11]. In June 2022, Serbia integrated the nine-valent HPV vaccine into its National Immunization Program, targeting both genders aged 9–19 years [12]. Additionally, in 2023, Bosnia and Herzegovina introduced HPV vaccination, with varying programs implemented across its constituent entities [13,14].

Several studies have investigated the influence of various socioeconomic factors and the quality of health services on cervical cancer epidemiology [15,16]. These studies highlight that in developing countries, lower cancer survival and higher mortality rates may be due to a higher percentage of late-stage cancer diagnoses, largely attributable to the lack of effective cervical cancer screening programs [15]. One such study from Northern France demonstrated that women from deprived areas are less likely to seek gynecological care compared to those from more affluent areas [16]. In fact, a study from Estonia assessed the impact of different sociodemographic factors on cervical cancer, where risk was increased among women who were younger, living in more remote regions, lower-educated, or divorced/widowed. Interruption in health insurance was associated with a 23% risk increase [17]. In the mentioned study, some regional differences in cervical cancer risk were also observed among screened women. Additionally, a study from Zreik and colleagues highlighted significant disparities in disease progression at diagnosis and survival for cervical cancer patients based on race/ethnicity and household income; lower household income was associated with poorer survival for stage [18].

Understanding factors that potentially affect cervical cancer rates is important for effective prevention and intervention measures. Identifying these factors could help us to tailor strategies to decrease incidence and mortality rates of cervical cancer, ultimately improving public health outcomes. This knowledge could enable healthcare providers to design targeted programs, allocate additional resources efficiently, and implement health policies that address the specific needs of different populations, ensuring comprehensive and equitable cancer prevention efforts.

The aim of this study was to analyze the epidemiology of cervical cancer in Serbia and neighboring Bosnia and Herzegovina, over a 5-year period, from January 2016 to December 2020, and prior to a wider implementation of the HPV vaccine in the national immunization programs of these two countries. Additionally, the study aimed to explore the relationship between the available sociodemographic factors and healthcare service parameters across administrative units, i.e., female population aged ≥15 years, area, population density, percent of gynecologists in primary healthcare according to plan, number of gynecologists per 10,000 females aged ≥15 years, and the area-to-gynecologist ratio, and the incidence and mortality rates.

## 2. Materials and Methods

### 2.1. Country Characteristics

Serbia and Bosnia and Herzegovina are neighboring countries located in the Balkan region of southeastern Europe. Serbia covers an area of 88,499 km^2^ and is divided into 25 administrative units. According to the most recent data before the period under investigation (2011 Census), the population of Serbia was 7,186,862 (without data from the Autonomous Province Kosovo and Metohija) [19,20]. With a real GDP per capita of USD 26,074 in 2023, the economy of Serbia is ranked 38^th^ out of 48 European states, below most neighboring countries except Bosnia and Herzegovina, North Macedonia, and Albania [21]. Bosnia and Herzegovina shares a border with Serbia to the east, Montenegro to the southeast, and Croatia to the southwest, west, and north [8]. The country is highly decentralized, consisting of two constitutive entities, the Federation of Bosnia and Herzegovina (FB&H) and the Republic of Srpska (RS), and the Brčko District. The FB&H is subdivided into 10 administrative units—cantons—while the RS is presented as a single unit, as it is not divided into administrative units according to its Constitution. The total area of Bosnia and Herzegovina is 51,209 km^2^, and the estimated population is 3,434,000. With a real GDP per capita of USD 19,634, Bosnia and Herzegovina ranks 43^rd^ out of 48 European states, making it poorer than all neighboring countries [8,22].

### 2.2. Data Collection

We used an ecological approach based on aggregated data. Data related to cervical cancer epidemiology for the period 2016–2020 and healthcare services were obtained from the annual publications of the Institute of Public Health of Serbia “Dr Milan Jovanović Batut” [23], the Institute for Public Health of the Federation of Bosnia and Herzegovina [24,25,26], and the National Institute for Statistics and Public Health Institute of the Republic of Srpska [27,28]. Data from the Public Health Subdivision of the Department of Health and Other Services of Brčko were excluded from the analyses due to a lack of standardization for incidence and mortality rates of cervical cancer. For demographic and territorial information, we used publicly available data from the Statistical Office of the Republic of Serbia [20], the Agency for Statistics of Bosnia and Herzegovina, the Federal Bureau of Statistics [22,24], and the Republic Statistical Institute in the Republic of Srpska [27].

Cervical cancer incidence and cervical cancer mortality by administrative units were extracted from the reports [23,24,25,26,27] and these rates were standardized by the method of direct standardization with the World-ASR-W [29]. In brief, cervical cancer screening in the two analyzed countries is prescribed and performed by gynecologists, utilizing the PAP test. In Serbia, organized screening has been conducted since 2012, by offering participation to all eligible women aged 25 to 69 years, including active invitation to screening, and the interval or frequency of screenings is every three years [30], while in Bosnia and Herzegovina, there is no active invitation for cervical screening for women aged 25–60, and the recommended interval for cytology screening is the same [31].

The following sociodemographic and healthcare service data were uniformly collected across the administrative units of both countries: female population aged ≥15 years, administrative unit’s area (in km^2^), population density (per km^2^), number of gynecologists per 10,000 females aged ≥15 years. Additionally, before proceeding to the data analyses, the percent of gynecologists according to plan (%) was calculated by dividing the number of employed gynecologists by the number of needed gynecologists as defined in the national regulations about the detailed conditions for performing healthcare activities in healthcare institutions and other forms of healthcare services [32,33,34]. Also, the area-to-gynecologist ratio was calculated by dividing the number of gynecologists in a specific district by the district’s area in square kilometers. This metric provides an indicator of gynecologist density, allowing for an assessment of healthcare accessibility relative to geographic size.

### 2.3. Statistical Analyses

To check for normality of the data distribution, we used the Shapiro–Wilk test and a visual inspection of the distribution plots. We used the Mann–Kendall trend test to analyze trends of the reported incidence and mortality rates over years and by country. The comparison of the values of numerical features between the groups was performed using the Kruskal–Wallis equality-of-populations rank test with Dunn’s multiple-comparison post hoc test for stochastic dominance and using the Bonferroni correction where appropriate. Numerical characteristics are presented as median values with the corresponding interquartile range (IQR). Administrative units, for the purpose of statistical analyses, were distributed into tertiles based on the values of the average incidence and mortality rates, where the first tertile contained units with the lowest and the third represented those with the highest values. Spearman’s rank correlation was used to explore potential correlations between values of different sociodemographic characteristics and health service usage of the administrative units, and the average incidence and mortality rates. Generalized linear regression models were fitted to examine potential predictors of incidence and mortality level across administrative units of the included countries. Statistical software STATA v.17 (College Station, TX, USA: StataCorp LLC 2021) was used for statistical data processing and analyses. Values of *p* < 0.05 were considered statistically significant.

## 3. Results

According to the estimates for 2018, the total female population aged ≥15 years in Serbia was approximately 3.1 million, in the FB&H was 960,000, and in the RS 547,000. Population density was 78.9 inhabitants per km^2^ in Serbia, 84.1 in the FB&H, and 46.6 in the RS. The numbers of gynecologists in primary healthcare were 1.44, 1.03, and 2.03 per 100,000 females aged ≥15 years in Serbia, the FB&H, and the RS, respectively. The ratio of country area (in km^2^) to the number of gynecologists was 198.43 km^2^ per gynecologist in Serbia, 263.74 in the FB&H, and 221.99 in RS. The average age-standardized incidence rate of cervical cancer during 2016–2020 was 19.28 per 100,000 females in Serbia, 12.48 in the Federation of Bosnia and Herzegovina, and 22.44 in the Republic of Srpska, while the average age-standardized mortality rate of cervical cancer for the same time period was 6.67, 5.22, and 4.56 per 100,000 females in Serbia, the FB&H, and RS, respectively, as presented in Table 1.

**Figure 1:**
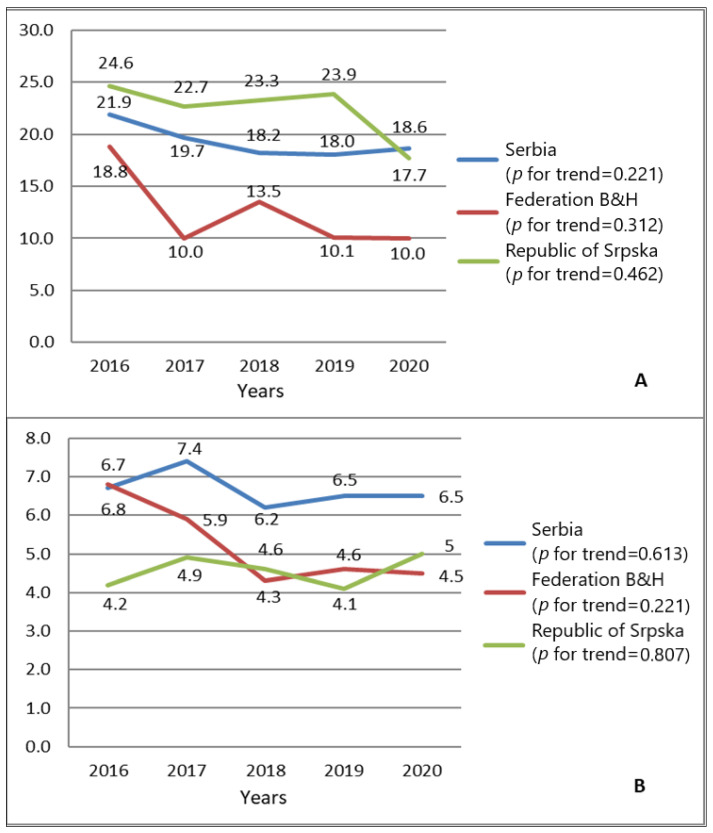
Standardized incidence (**A**) and mortality (**B**) rates of cervical cancer in Serbia, the Federation of Bosnia and Herzegovina, and the Republic of Srpska (2016–2020). Note: the Mann–Kendall trend test was used to analyze trends.

During the observed period (2016–2020), the highest incidence was recorded in 2016 in the RS (24.6 per 100,000 females), followed by Serbia and the FB&H (21.9 and 18.8 per 100,000 females, respectively). The lowest incidence was reported in 2017 and 2020 in the Federation of Bosnia and Herzegovina (10.0 per 100,000 females), in Serbia in 2019 (18.0 per 100,000 females), and in the Republic of Srpska in 2020 (17.7 per 100,000 females). Mortality rates were the highest in 2020 for the RS (5.0 per 100,000 females), in 2017 for Serbia (7.4 per 100,000 females), and in 2016 in the FB&H (6.8 per 100,000 females). The lowest mortality rates were reported in 2018 for the Federation of Bosnia and Herzegovina (4.3 per 100,000 females) and Serbia (6.2 per 100,000 females), and in 2019 for the Republic of Srpska (4.1 per 100,000 females) (Figure 1).

**Figure 2:**
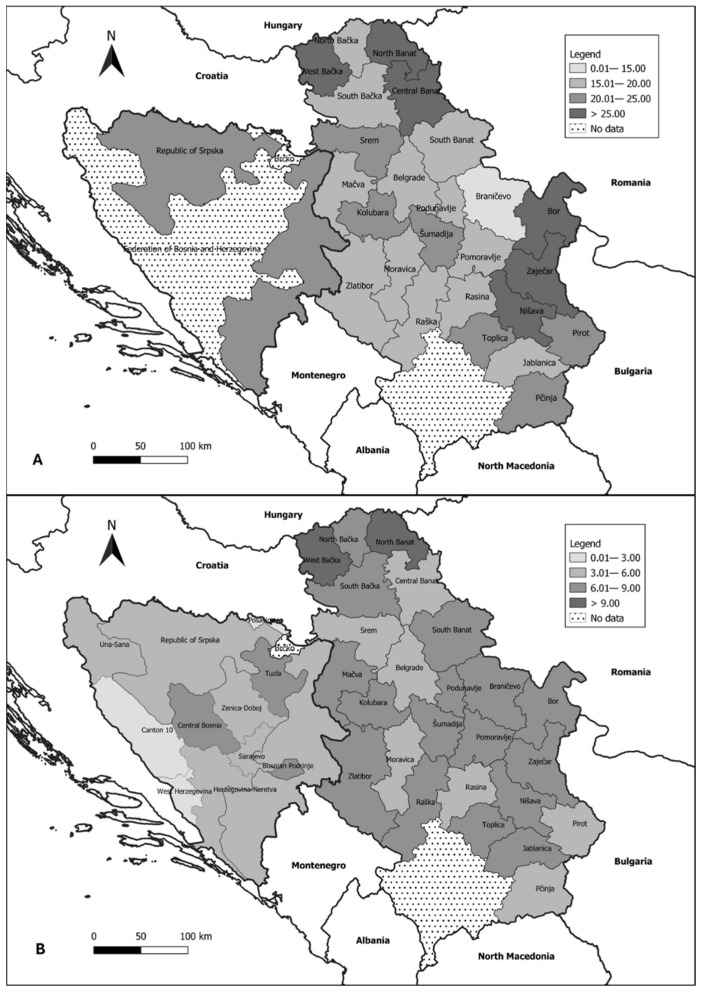
Standardized incidence (**A**) and mortality (**B**) rates of cervical cancer in Serbia, the Federation of Bosnia and Herzegovina, and the Republic of Srpska by the administrative units (2016–2020). Note: The Republic of Srpska is not divided by administrative units according to the constitution and is presented as a single unit.

We further analyzed incidence and mortality rates across the administrative units of Serbia and of Bosnia and Herzegovina, as illustrated in Figure 2. We observed that the peripheral administrative units of Serbia reported the highest average incidence rates: Bor District in the east (31.76 per 100,000 females), West Bačka District in the northwest (28.38 per 100,000 females), and Central Banat District in the northeast (27.78 per 100,000 females). Conversely, the central regions reported the lowest average incidence rates: Braničevo District (14.54 per 100,000 females) and Rasina District (16.38 per 100,000 females). In Bosnia and Herzegovina, the Republic of Srpska (not subdivided into administrative units) had an average incidence rate of 22.44 per 100,000 females, while detailed incidence data for the ten administrative units of the Federation of Bosnia and Herzegovina were not available. In Serbia, the highest mortality rates were observed in North Banat (10.30 per 100,000 females) and West Bačka District (9.32 per 100,000 females), whereas the lowest rates were reported in Moravica District (5.16 per 100,000 females). In Bosnia and Herzegovina, Tuzla Canton had the highest reported mortality rate at 6.98 per 100,000 females, while Canton 10 had the lowest reported rate of 0.12 per 100,000 females.

We divided administrative units of the included countries into three groups based on tertiles of incidence and mortality rates in order to explore differences in sociodemographic factors and healthcare service parameters across these groups—namely those with the lowest, medium, and the highest reported values of incidence and mortality rates (Table 2). For the average incidence across 26 administrative units in the two countries, the median incidence in the first tertile was 16.98 (IQR = 16.84–17.36), in the second tertile was 20.04 (18.26–20.18), and in the third tertile was 25.81 (IQR = 24.62–28.08). We observed a statistically significant difference across tertile incidence groups in the values of total female population ≥ 15 years old (*p* = 0.028), population density (*p* = 0.046), percent of gynecologists in the primary healthcare (*p* = 0.041), number of gynecologists per 10,000 females ≥ 15 years (*p* = 0.007), and the area-to-gynecologist ratio (*p* = 0.010).

On the other hand, when we categorized administrative units by reported mortality rates, those units in the first tertile had the lowest values (median = 4.86, IQR = 2.72–5.41), followed by the second tertile (median = 6.28, IQR = 6.10–6.59), and the third tertile, which included units with the highest mortality rates (median = 7.59, IQR = 7.29–8.30). None of the explored variables demonstrated statistically significant differences across the mortality tertile groups.

We explored correlations between the selected sociodemographic characteristics and healthcare service parameters with the average incidence and mortality rates across administrative units of the included countries, and the results are presented in Table 3 and Supplementary Figure S1. We observed a statistically significant moderate negative correlation between incidence rate and a unit’s population density (rho = −0.465, *p* = 0.017), as well as a moderate positive correlation between incidence rate and the area-to-gynecologist ratio (rho = 0.534, *p* = 0.005).

**Figure 3:**
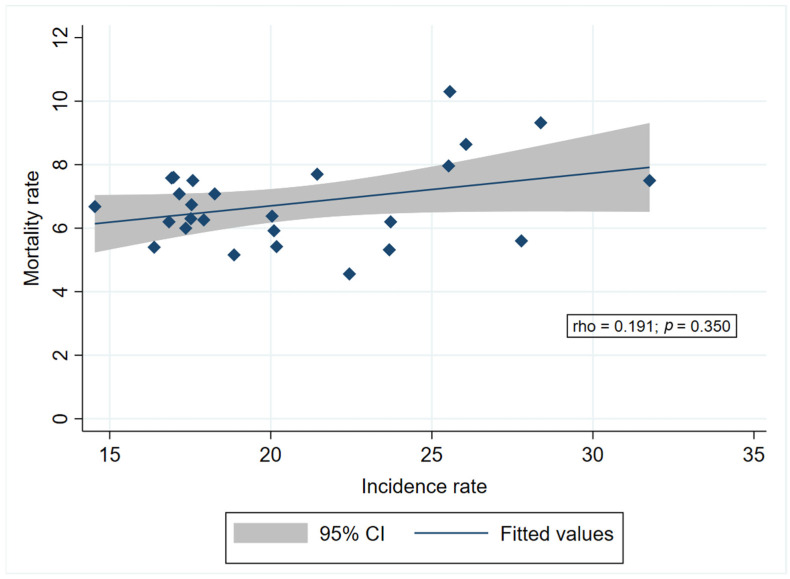
Correlation between the average incidence and the average mortality rates of cervical cancer across administrative units of the included countries (2016–2020). Note: each diamond represents one administrative unit.

Additionally, Figure 3 shows a weak positive correlation between average incidence and average mortality rates of cervical cancer across the included administrative units, although this correlation is not statistically significant (rho = 0.191, *p* = 0.350).

Finally, we examined potential predictors of cervical cancer incidence and mortality rates across administrative units using the univariate analysis. We found that each unit increase in the area-to-gynecologist ratio was associated with an estimated increase of 0.0007 in the average incidence rate (exp(b) = 1.000693, 95% CI: 1.000271–1.001114, *p* = 0.001). Similarly, it was a modest predictor of the mortality rate, with each unit increase in the area-to-gynecologist ratio corresponding to a 0.0007 decrease in the average mortality rate (exp(b) = 0.9993034, 95% CI: 0.9986751–0.9999321, *p* = 0.030) (Table 4). When introducing a weighting variable in the model, by adding the number of female populations aged ≥15 years, the results remained stable, i.e., the only parameter that was additionally found to be a significant predictor was the percent of gynecologists in primary healthcare according to the plan, where each unit increase in this parameter corresponded to a 0.004 decrease in the average incidence rate (adj. exp(b) = 0.9964476, 95% CI: 0.9932942–0.9996111, *p* = 0.028) (Table S1).

## 4. Discussion

During 2016–2020, the average age-standardized incidence rate of cervical cancer was 19.28 in Serbia, 12.48 in the Federation of Bosnia and Herzegovina, and 22.44 per 100,000 females in the Republic of Srpska, while the average age-standardized mortality rate of cervical cancer was 6.67, 5.22, and 4.56 per 100,000 females in Serbia, the Federation of Bosnia and Herzegovina, and the Republic of Srpska, respectively. Significant differences were noticed across incidence tertile groups in the values of total female population ≥ 15 years old, population density, percent of gynecologists in primary healthcare, number of gynecologists per 10,000 females ≥ 15 years, and the area-to-gynecologist ratio, while across the mortality tertile groups none of the explored variables demonstrated significant differences. Incidence rate and unit’s population density showed a moderate negative correlation, while there was a moderate positive correlation between incidence rate and the area-to-gynecologist ratio. Finally, area-to-gynecologist ratio was found to be a modest predictor of the incidence and mortality rate.

Cervical cancer incidence and mortality rates vary significantly across different geographical regions. They are higher in underdeveloped areas (e.g., Sub-Saharan African and South-Eastern Asian countries), primarily due to limited healthcare infrastructure, lack of screening programs (or their inefficient implementation), and minimal access to HPV vaccination, contributing to very high incidence and mortality rates of cervical cancer [7]. On the contrary, economically developed countries (e.g., Western Europe and North America) experience a lower burden of cervical cancer [7]. These countries typically have robust healthcare systems with widespread access to screening programs (such as Pap smears) and HPV vaccination. Developing countries, including Serbia and Bosnia and Herzegovina, fall in between, experiencing a moderate to high burden of incidence (age-standardized incidence rate of 26.6 and 21.42 per 100,000) and mortality (9.4 vs. 4.5/100,000) of cervical cancer [4,6]. The incidence rate of cervical cancer per district is an important indicator, reflecting both risk factors and the effectiveness of screening programs within the population. Conversely, cervical cancer mortality rates provide insight into treatment outcomes and the effectiveness of management strategies within each district. Our results indicate that, over a five-year period, the average age-standardized incidence rate in Serbia and Bosnia and Herzegovina was far higher than the estimated rate for Southern Europe (7.72 per 100,000 females) [4,6]. Although the average age-standardized incidence rate of cervical cancer in the Federation of Bosnia and Herzegovina during the observed period was 12.48 per 100,000, which is relatively better compared to other regions from our research, it remains above the average incidence rates reported in other European countries [4]. In specific, mortality rates in Serbia are significantly higher compared to those in Bosnia and Herzegovina (RS and the FB&H). Limited healthcare resources in these countries may present challenges in implementing comprehensive cervical cancer prevention and control measures. In general, poverty affects health behaviors and outcomes, with all impoverished communities facing economic vulnerability due to unstable employment and limited access to quality services. High incidence and mortality from cancer is a characteristic of high-poverty communities, regardless of rural or urban setting [35]. In-depth analysis of healthcare resources should be undertaken to have a complete understanding of their influence on the incidence and mortality rates in these countries. Also, screening programs might be implemented sporadically, and access to HPV vaccination can be limited, particularly in rural or underserved areas [4]. Despite the global use of the HPV vaccine for over 15 years, its implementation in Serbia and Bosnia and Herzegovina has only recently begun [12,13,14,36,37]. Even with the proven benefits of well-organized screening, there are still districts and municipalities in both countries where cervical cancer screening remains opportunistic.

The number of gynecologists per 10,000 females aged ≥15 years indicates the availability of specialized gynecological care relative to the female population aged ≥15 years. This metric is important for assessing healthcare accessibility and resource allocation. Additionally, population density is a useful demographic factor affecting healthcare delivery. Higher density often correlates with increased demand for healthcare services, including gynecological care [38]. The geographic size of a district (in km^2^) can impact the provision of healthcare services, particularly in rural or sparsely populated areas, where larger areas may present logistical challenges for both providers and patients. Considering these factors, we analyzed selected health system variables and their potential influence on incidence and mortality rates in Serbia and Bosnia and Herzegovina. Although these variables are not necessarily directly causally related to the observed incidence or mortality rates, our findings indicate a positive correlation between the area-to-gynecologist ratio and the incidence rates of cervical carcinoma in different administrative units across Serbia and Bosnia and Herzegovina. Specifically, larger areas covered by a single gynecologist were associated with higher incidence rates of cervical carcinoma. These results are consistent with numerous studies highlighting the critical importance of the availability and accessibility of gynecologists for conducting regular gynecological examinations [38,39,40]. It can be that, besides the availability of gynecologists, a low level of awareness and a low uptake of cervical cancer screening in this population influences the observed incidence and mortality rates, but these data were not available to us. A recent study by Srinath et al. examined barriers to cervical cancer screening by reviewing 67 articles from low- and middle-income countries. They used a framework focusing on accessibility, identifying five key aspects: approachability, acceptability, availability, affordability, and appropriateness. Their findings confirmed that lack of awareness about the importance of preventive programs, the cost of screening services, and the distance to screening centers were major barriers [39]. These factors may explain our findings related to the variable area-to-gynecologist ratio and increased incidence. It is crucial for policymakers and governments to take additional steps to build public confidence in health systems. Women should be educated about cancer causes and risk factors through evidence-based strategies in order to enhance adherence to screening programs. A Japanese study by Yu et al. aimed to quantify the effect of screening tests on cervical cancer incidence and concluded that a 10% increase in screening test rate is estimated to effectively decrease cervical cancer incidence by 9.6% and that additional countermeasures for prevention are needed [41].

Different areas in Serbia and Bosnia and Herzegovina exhibit significant variation in the availability of healthcare providers for women, particularly in rural and inaccessible regions. This disparity is especially critical for preventive examinations of insidious diseases such as cervical cancer, where symptoms may remain absent for many years and even decades. A previous study indicates that the lack of healthcare facilities providing screening services in rural areas is a major barrier. Women often have to travel long distances or invest considerable time and money to reach a screening center, which significantly impedes access to gynecological services [38]. In Serbia, cervical cancer screening has been conducted since 2012, utilizing the Pap test, which is based on the cytomorphological examination of cervical samples. Although screening is offered to all eligible women aged 25 to 69 years, it is not uniformly implemented across the entire country due to logistical challenges, resulting in varying levels of coverage. As a result, cervical cancer remains one of the most common cancers among women in Serbia. In Bosnia and Herzegovina, there is no active invitation for cervical screening for women aged 25–60, and the recommended interval for cytology screening is every three years [31]. It can be assumed that many rural areas in these two neighboring countries face issues with accessibility to medical facilities (doctor’s offices). A study from France revealed that, in a multivariable analysis, the European Deprivation Index (EDI) of the doctor’s office was strongly associated with the cervical cancer screening participation rate of eligible patients. The EDI related to the location of the family doctor’s office appears to be a robust predictor of female patients’ participation in cervical cancer screening [16].

Well-planned screening programs with high coverage of women at the district or country level can reduce cervical cancer mortality rates [15]. In our study, we found that the area-to-gynecologist ratio was a modest predictor of mortality rate. This finding may be explained by the fact that the number of gynecologists in a region does not necessarily reflect women’s awareness of preventive procedures or the actual state of women’s health in that specific region.

Public health measures to reduce inequalities and improve healthcare, such as increasing preventive services and investing in healthcare infrastructure across the administrative units, could impact cervical cancer rates [16,17]. Additionally, educational activities on reproductive health, and offering HPV vaccines to women up to 45 years of age, regardless of previous contact with HPV, could help reduce future cervical cancer rates [17]. The varying cervical cancer incidence rates in Serbia and Bosnia and Herzegovina may result from several factors, including disparities in access to preventive healthcare services, sociodemographic differences, varying public health policies, and cultural influences. These observed differences likely represent a combination of various factors, emphasizing the need for targeted interventions and tailored public health strategies for each specific region [16,17,18].

Our research has several limitations that should be considered when interpreting the results. We used population-level data, which prevented us from exploring relationships between the studied variables. Due to a slightly different approach in data collection and presentation of some sociodemographic parameters across two countries, we limited our analyses to just a few parameters uniformly presented. Additionally, as previously mentioned, the Brčko District, the third constitutive entity of Bosnia and Herzegovina, did not provide standardized rates, so we were unable to present a comprehensive overview of the epidemiological situation in Bosnia and Herzegovina, even though this entity represents a relatively small portion (around 2.5%) of the population in Bosnia and Herzegovina. Also, we did not have access to personal data related to the nationalities of patients and their health behaviors, which could contribute to the incidence of the observed disease. Furthermore, we lack data on the cancer stage at diagnosis and applied therapy, which could have influenced cancer survival and reported mortality rates. Since cervical cancer screening is not conducted uniformly across administrative units of the two countries and these data were not available to us on an administrative unit level, we used the number of gynecologists as a proxy of the availability and distance of gynecological service. More research is needed to further investigate the uptake and participation in the screening programs, health awareness, and potential barriers in accessing these services among women in the region.

## 5. Conclusions and Public Health Implications

Developing countries often face numerous challenges, particularly concerning the functioning of their healthcare systems. We found that the area-to-gynecologist ratio significantly increased from administrative units with the lowest to the highest incidence, while population density decreased from the lowest to the highest incidence group. Also, we reported a moderate negative correlation between incidence rate and population density as well as a moderate positive correlation between incidence and area-to-gynecologist ratio. The area-to-gynecologist ratio was a significant predictor of average incidence and mortality rates.

Explored variables collectively provide an overview of gynecological health in two countries, facilitating targeted interventions and resource allocation to improve health outcomes. It underscores the importance of integrated health service planning and policy-making. Cervical cancer remains a leading cancer among women in developing countries like Serbia and Bosnia and Herzegovina, and a strong response from the public health sector is warranted to reduce inequalities and to improve healthcare and outcomes for cervical cancer.

## Figures and Tables

**Table 1:** General description of the analyzed sociodemographic factors and healthcare service parameters across the included countries.

	Serbia	Bosnia and Herzegovina ^a^	
		Federation of Bosnia and Herzegovina (FB&H)	Republic of Srpska (RS)
Female population aged ≥15 years	3,095,721	960,075	547,799
Area (in km^2^)	88,499	26,110	24,641
Population density (per km^2^)	78.9	84.1	46.6
Percent of gynecologists according to plan (%)	93.6	102.1	67.7
Gynecologists per 10,000 females, aged ≥15 years	1.44	1.03	2.03
Area-to-gynecologist ratio (in km^2^)	198.43	263.74	221.99
Cervical carcinoma standardized incidence rate (average per 100,000)	19.28	12.48	22.44
Cervical carcinoma standardized mortality rate (average per 100,000)	6.67	5.22	4.56

^a^ Data for District Brčko in Bosnia and Herzegovina were not available.

**Table 2:** Selected sociodemographic factors and healthcare service parameters across administrative units of Serbia and Bosnia and Herzegovina based on the tertiles of cervical cancer incidence and mortality rates (2016–2020).

	Administrative Units Distributed by Incidence Rate (n = 26) ^a^				Administrative Units Distributed by Mortality Rate (n = 36)			
	First Tertile Group (n = 9)	Second Tertile Group (n = 9)	Third Tertile Group (n = 8)	*p*- Value ^b^	First Tertile Group (n = 12)	Second Tertile Group (n = 12)	Third Tertile Group (n = 12)	*p*- Value ^b^
Female population aged ≥15 years, median (IQR)	**99,626 (81,859–** **122,327)**	**117,136 (88,775–** **126,994)**	**66,436 (50,035–** **77,556)**	**0.028 ^c^**	92,338 (38,435.5– 123,652.5)	112,172 (78,086– 157,343.5)	84,493 (55,974– 124,529.5)	0.579
Area (in km^2^), median (IQR)	3234 (2668– 3857)	3485 (2614– 3923)	2744.5 (2481– 3382)	0.567	3300.5 (2714.5– 4263)	3211.5 (2086– 3941.5)	2688.5 (2408– 3388.5)	0.430
Population density (per km^2^), median (IQR)	**85.72** **(72.57–** **149.60)**	**66.38** **(46.59–** **77.78)**	**56.37** **(31.47–** **67.89)**	**0.046 ^d^**	65.83 (48.03– 84.92)	72.40 (51.12– 136.10)	74.48 (48.29– 109.27)	0.544
Percent of gynecologists in primary healthcare according to plan (%), median (IQR)	**90.33** **(84.53–** **95.85)**	**113.99 (99.88–** **126.49)**	**76.19** **(65.15–** **93.13)**	**0.041 ^e^**	82.68 (71.34– 113.24)	87.43 (72.68– 124.99)	96.44 (87.27– 112.25)	0.846
Gynecologists per 10,000 females aged ≥15 years, median (IQR)	**1.39** **(1.30–1.47)**	**1.77** **(1.73–2.03)**	**1.17** **(1.00–1.43)**	**0.007 ^f^**	1.21 (0.93–1.63)	1.26 (1.05–1.76)	1.47 (1.20–1.73)	0.613
Area-to-gynecologist ratio (in km^2^), median (IQR)	**184.67 (122.00–** **205.23)**	**195.56 (163.38–** **274.18)**	**373.93 (301.00–** **454.06)**	**0.010 ^g^**	299.09 (213.61– 432.73)	223.99 (106.92– 347.27)	183.17 (144.76– 314.86)	0.182
Incidence rate per 100,000 females (2016–2020 average), median (IQR)	16.98 (16.84– 17.36)	20.04 (18.26– 20.18)	25.81 (24.62– 28.08)	NA	21.31 (18.86– 23.68)	17.54 (17.36– 20.04)	21.44 (17.16– 26.06)	0.196
Mortality rate per 100,000 females (2016–2020 average), median (IQR)	6.68 (6.20–7.08)	6.26 (5.42–7.08)	7.73 (5.90–8.98)	0.247	4.86 (2.72–5.41)	6.28 (6.10–6.59)	7.59 (7.29–8.30)	NA

^a^ For ten administrative units in the Federation of Bosnia and Herzegovina, detailed incidence data were not available. ^b^ Using Kruskal–Wallis equality-of-populations rank test with Dunn’s multiple-comparison post hoc test for stochastic dominance using a Bonferroni correction where appropriate. ^c^ First vs. third tertile group, *p* = 0.033; second vs. third tertile group *p* = 0.026. ^d^ First vs. third tertile group, *p* = 0.022. ^e^ Second vs. third tertile group, *p* = 0.018. ^f^ First vs. second tertile group, *p* = 0.047; second vs. third tertile group, *p* = 0.004. ^g^ First vs. third tertile group, *p* = 0.006; second vs. third tertile group, *p* = 0.037. n = number. NA = not applicable. In bold are significant results at *p* < 0.05.

**Table 3:** Correlation between administrative unit’s characteristics and cervical cancer incidence and mortality rates across administrative units of Serbia and Bosnia and Herzegovina (2016–2020).

	Incidence Rate Across Administrative Units (n = 26) ^a^		Mortality Rate Across Administrative Units (n = 36)	
	Spearman’s Rho	*p*-Value ^b^	Spearman’s Rho	*p*-Value ^b^
Female population aged ≥15 years	−0.361	0.070	0.005	0.977
Area (in km^2^)	−0.030	0.883	−0.186	0.277
Population density (per km^2^)	**−0.465**	**0.017**	0.068	0.695
Percent of gynecologists in primary healthcare according to plan (%)	−0.268	0.186	0.017	0.923
Gynecologists per 10,000 females aged ≥15 years	−0.179	0.382	0.150	0.382
Area-to-gynecologist ratio (in km^2^)	**0.534**	**0.005**	−0.211	0.216

^a^ For ten administrative units in the Federation of Bosnia and Herzegovina, detailed incidence data were not available. ^b^ Using Spearman’s rank correlation coefficient. In bold are significant results at *p* < 0.05.

**Table 4:** Predictors of the cervical cancer incidence and mortality level across administrative units of the included countries (2016–2020).

	Incidence Rate Across Administrative Units (n = 26) ^a^		Mortality Rate Across Administrative Units (n = 36)	
	exp(b) (95% CI) *	*p*-Value	exp(b) (95% CI) *	*p*-Value
Female population aged ≥15 years	0.9999998 (0.9999992–1.000000)	0.331	1.000000 (0.9999992–1.000001)	0.996
Area (in km^2^)	1.000002 (0.9999826–1.000022)	0.819	0.9999864 (0.9999569–1.000016)	0.368
Population density (per km^2^)	0.9993638 (0.9985404–1.000188)	0.130	1.000101 (0.9988376–1.001365)	0.876
Percent of gynecologists in primary healthcare according to plan (%)	0.996916 (0.9937315–1.000111)	0.058	0.9996847 (0.9951569–1.004233)	0.892
Gynecologists per 10,000 females aged ≥15 years	0.8416694 (0.6839407–1.035773)	0.104	1.207599 (0.8980568–1.623835)	0.212
Area-to-gynecologist ratio (in km^2^)	**1.000693** **(1.000271–1.001114)**	**0.001**	**0.9993034** **(0.9986751–0.9999321)**	**0.030**
Incidence rate per 100,000 females (2016–2020 average)	NA	NA	1.014512 (0.9986296–1.030647)	0.074

^a^ Data for District Brčko in Bosnia and Herzegovina were not available. 95% CI = 95% confidence interval. NA—not applicable. * Using generalized linear model with gamma-distributed dependent variable and a log link function. In bold are significant results at *p* < 0.05.

## Data Availability

The data that support the findings of this study are available from the corresponding author upon reasonable request.

## Supplementary Materials

The following supporting information can be downloaded at https://www.mdpi.com/article/10.3390/medicina60121987/s1: Figure S1: Correlations between the average incidence rates and population density (A) and area-to-gynecologist ratio (B) across administrative units (2016–2020). Table S1: Results of the adjusted generalized linear model of the administrative unit’s predictors of the cervical cancer incidence and mortality level across administrative units of the included countries (2016–2020).
