# Supplementary material for: Relationship Between Population Density, Availability of Gynecological Services, and Cervical Cancer Incidence and Mortality Across Administrative Units in Serbia and Bosnia and Herzegovina During 2016–2020
Source: Medicina (Kaunas). 2024 Dec 2;60(12):1987. doi: 10.3390/medicina60121987 (PMC11727680; doi:10.3390/medicina60121987)
Supplement: Supplementary file 1 [file medicina-60-01987-s001.zip › medicina-3282235-supplementary.pdf]

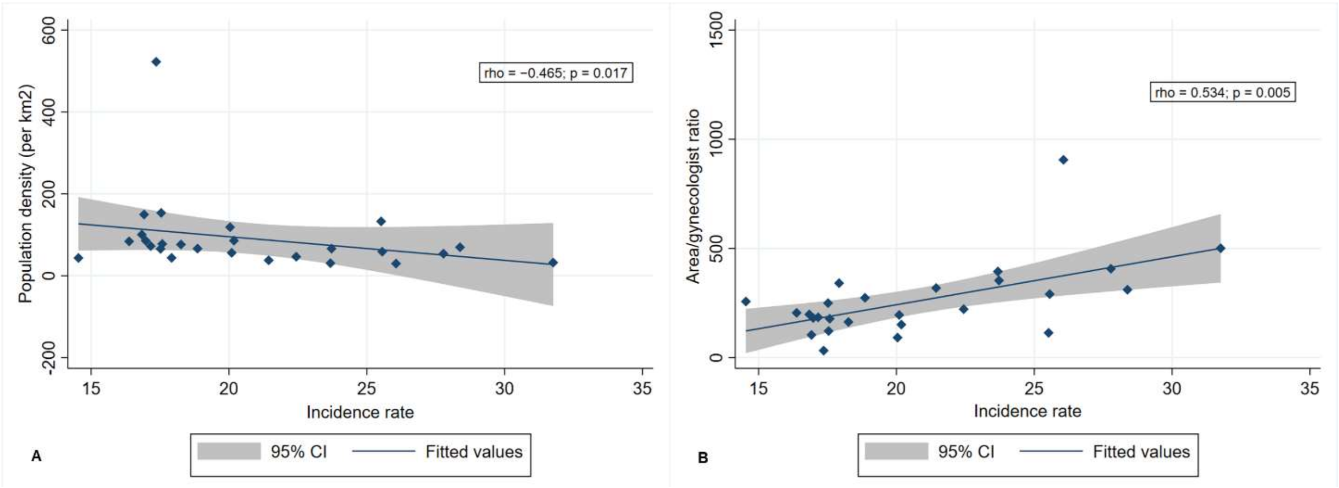

Figure S1. Correlation plots between the average incidence rates and population density (A) and area-to-gynecologist ratio (B) across administrative units, 2016-2020.

Table S1. Results of the adjusted generalized linear model of the administrative unit's predictors of the cervical cancer incidence and mortality level across administrative units of the included countries, 2016-2020.

	**Incidence rate across administrative units (n=26)[a]**		**Mortality rate across administrative units (n=36)**	
	**Adj. exp(b) (95% CI)***	**p-value**	**Adj. exp(b) (95% CI)***	**p-value**
Female population aged ≥15 years	-	-	-	-
Area (in km^2)	1.00001 (0.9999879-1.000033)	0.369	0.9999817 (0.9999454-1.000018)	0.322
Population density (per km^2)	0.9992465 (0.9978593-1.000636)	0.288	1.000241 (0.9983068-1.002179)	0.807
Percent of gynecologists in primary healthcare according to plan (%)	**0.9964476 (0.9932942-0.9996111)**	**0.028**	0.9996825 (0.9950777-1.004309)	0.893
Gynecologists per 10,000 females aged ≥15 years	0.849806 (0.6887309-1.048552)	0.129	1.208913 (0.8947266-1.633426)	0.217
Area-to-gynecologist ratio (in km^2)	**1.000713 (1.000242-1.001184)**	**0.003**	**0.9991775 (0.99849-0.9998656)**	**0.019**
Incidence rate per 100,000 females (2016-2020 average)	NA	NA	1.012066 (0.9961121-1.028275)	0.139

[a]Data for District Brčko in B&H were not available. 95% CI= 95% confidence interval. NA- not applicable. *Using generalized linear model with gamma-distributed dependent variable and a log link function and adjusted for a weight variable, i.e., female population aged ≥15 years. In bold are significant results at $p < 0.05$.
